# Analyzing repeated data collected by mobile phones and frequent text messages. An example of Low back pain measured weekly for 18 weeks

**DOI:** 10.1186/1471-2288-12-105

**Published:** 2012-07-23

**Authors:** Iben Axén, Lennart Bodin, Alice Kongsted, Niels Wedderkopp, Irene Jensen, Gunnar Bergström

**Affiliations:** 1 Institute of Environmental Medicine, Unit of Intervention and Implementation Research, Karolinska Institutet, Nobels v. 13, 171 77, Stockholm, Sweden; 2 Nordic Institute of Chiropractic and Clinical Biomechanics, Clinical Locomotion Network, Forskerparken 10A, 5230, Odense M, Denmark; 3 Institute of Regional Health Services Research, University of Southern Denmark, Winsloewparken 19.3, 5000, Odense, Denmark

## Abstract

**Background:**

Repeated data collection is desirable when monitoring fluctuating conditions. Mobile phones can be used to gather such data from large groups of respondents by sending and receiving frequently repeated short questions and answers as text messages.

The analysis of repeated data involves some challenges. Vital issues to consider are the within-subject correlation, the between measurement occasion correlation and the presence of missing values.

The overall aim of this commentary is to describe different methods of analyzing repeated data. It is meant to give an overview for the clinical researcher in order for complex outcome measures to be interpreted in a clinically meaningful way.

**Methods:**

A model data set was formed using data from two clinical studies, where patients with low back pain were followed with weekly text messages for 18 weeks. Different research questions and analytic approaches were illustrated and discussed, as well as the handling of missing data. In the applications the weekly outcome “number of days with pain” was analyzed in relation to the patients’ “previous duration of pain” (categorized as more or less than 30 days in the previous year).

Research questions with appropriate analytical methods

1: *How many days with pain do patients experience?* This question was answered with data summaries.

2*: What is the proportion of participants “recovered” at a specific time point?* This question was answered using logistic regression analysis.

3: *What is the time to recovery?* This question was answered using survival analysis, illustrated in Kaplan-Meier curves, Proportional Hazard regression analyses and spline regression analyses.

4: *How is the repeatedly measured data associated with baseline (predictor) variables?* This question was answered using generalized Estimating Equations, Poisson regression and Mixed linear models analyses.

5: *Are there subgroups of patients with similar courses of pain within the studied population?* A visual approach and hierarchical cluster analyses revealed different subgroups using subsets of the model data.

**Conclusions:**

We have illustrated several ways of analysing repeated measures with both traditional analytic approaches using standard statistical packages, as well as recently developed statistical methods that will utilize all the vital features inherent in the data.

## Background

Information collected for clinical research is usually gathered from the participants using questionnaires (paper- or web-based) or diaries. Diaries are often used when several points of measure are of interest, in studying the progress or the development of a condition over time. In theory, this is an excellent method. However, studies have shown that respondents have a tendency to backfill entries [1], and thus diary measurements rely heavily on memory at the expense of data validity.

The term “Ecological Momentary Assessment” (EMA) has been used to describe the “repeated sampling of subjects’ current behaviors and experiences in real time in subjects’ natural environment” [2]. This was previously exclusive to diaries. Through the use of mobile phones, sending and retrieving information repeatedly and as frequently as requested to large groups of people through text messages is possible. In Sweden, 94% of the population owned a mobile phone in 2008 [3]. Further, most people seem to carry their phone with them at all times, thus making measurements truly ecological, i.e. taking place in the patients’ own environment, which may be important when context is influencing the variable of interest. Further, the measurements are momentary, and the use of mobile phones makes seasonal disruption (such as holidays) of data collection minimal [4].

The “SMS-Track Questionnaire” [5] is a software system utilizing this technology; automatic short text messages are sent to study subjects at any desired frequency. The system has been developed specifically for research, and the responses are immediately recorded in a data sheet which minimizes further data handling and thus risk of error during this process. The data may be accessed by the researcher in real time on the internet, and the system has been shown to be highly financially favourable compared to questionnaires [6]. Further, a previous study showed a high response rate [7] without any optimization measures, and other studies showed exceptionally high response rates with simple interventions such as providing initial information and calling to remind respondents who fail to answer [4,6]. The method has shown to be user friendly and to yield good compliance [4]. It has been used to evaluate the clinical course of low back pain (LBP)[4,6-8] and to detect sports injuries in children [9]. By means of repeated longitudinal data detailed prospective research regarding course, inception, recovery, exacerbation and periodicity is possible.

The analysis of such repeatedly collected data, regardless of collection tool, presents new challenges. Even when compliance is high, invariably most respondents will have some missing values when measured repeatedly over months or years. Further, as the data produced contain repeated measurements of the same individuals over time, within-subject correlation must be considered in the analysis. In addition, when considering patients, they will often come from different clinics, introducing one more level into the analysis. Finally, it is desirable to analyze the complex outcome measure so that it can be interpreted in a clinically meaningful way.

The overall aim of this commentary is to describe different methods of analyzing repeated data. An overview of different statistical methods were applied to a model data set based on actual data collected with text messages in two of the referenced studies [4,7]. The results were used as a basis for discussion of the challenges, possibilities and appropriateness of each method.

## Methods

### Participants and measurements

The data used to make the model data set were aggregated from two clinical studies, one Danish [7] and one Swedish [4]. By using data from two studies, the intent was to add diversity and to increase the number of highly compliant participants. From both cohorts, respondents with very low response rates (those answering one or two of the 18 weeks) were removed before merging the datasets. Both studies examined patients with low back pain (LBP) in chiropractic consultations. The Swedish study was approved by the Ethics committee at the Karolinska Institutet (2007/1458-31/4) and the Danish study was reviewed by the local ethics committee which stated that it did not need approval. A total of 244 patients were included in the model data set; 49% were male, the mean age was 43.5 years (SD 11.0) and 50% of the sample had had LBP for more than 30 days in the previous year. Throughout this commentary, we will use “previous duration” as the baseline characteristic of interest. Thus, the sample will be stratified into patients with short (< 30 days) and long (>30 days) duration of LBP in the previous year, and comparisons between these two groups will be made.

The patients in both studies were informed about the text message method, the exact wording of the text message question (asking about the number of days with bothersome LBP during the preceding week) as well as the answer options (a number between 0 and 7) at inclusion. In both studies patients were followed for 18 weeks with weekly text message questions.

It should be noted that this model data set represents a clinical situation in which patients initially report relatively high levels of pain. A treatment period follows, in which pain reduction is aimed for. Thus, the model data set is not a surveillance of patients in a steady or normal state of health, in which recurrence or inception of a condition would be of interest.

### Analytic approaches

The method of analysis depends mainly on the research question asked. In general terms, SMS-track data can be used as an outcome in variable oriented (group level) prognostic or effect studies. The analysis may also be person oriented [10], searching for subgroups with distinct pain patterns with small within-subgroup variation and high between-subgroup variation.

The method of analysis also depends on the data. Some analyses are suitable only for continuous data and not for categorical data. Further, data may have to be converted by a log or square root transformation to fit the assumption of normal distribution of the outcome.

Many methods of analysis assume independence of data and therefore are not suitable for repeated data. When a subject is measured repeatedly, the data from that individual (within-subject) are bound to show a stronger correlation compared to those between individuals (between-subject). Failure to account for this co-variance may result in inaccurate confidence intervals, that is, the nominal coverage of e.g. 95% will not be accomplished.

In this commentary, clinically relevant research questions are presented with an overview of some of the possible analytic approaches. We have treated the measure “number of pain days” as both a continuous and a count variable to illustrate the different methods of analysis. Each method is then discussed in relation to the results of the different subset analyses. The research questions 1–4 are variable-oriented (related to group level analyses), and question number 5 is person-oriented.

### Missing data

Repeated data collection invariably contains some missing data. Without special precautions many analytical methods for longitudinal data cannot directly handle individuals who have missing data for any of the time points and those individuals are simply excluded from analysis, which is known as listwise or casewise deletion. This might introduce bias when the follow up time of a study is long with many measurements, as most subjects will have some missing data, and missing data most often do not occur at random. Exclusion of individuals will, apart from the risk of bias, also lead to less efficient estimates, e.g. larger confidence intervals.

Imputation of missing data is an alternative to facilitate analysis of repeated measures. One of the earliest imputation techniques for longitudinal data is the “last value carried forward”, where the missing value is simply replaced by the previously recorded value. When studying a clinical course, this would probably be a crude but rather accurate action, as each individual value correlates the strongest with the measurements closest in time. However, over recent decades more elaborate ways of imputation of data have been developed, see Little and Rubin [11] for a review of methods. In particular, multiple imputation methods have been developed.

These methods aim to not only impute values but also to take into account the increased sampling variability due to the imputation of missing data. However, in the examples presented in this commentary, no imputations were done, but the problems of missing longitudinal data deserve special attention. A second approach to handling missing data is direct maximum likelihood (DML), see Enders [12] for a primer on this method and a comparison with multiple imputation.

Without imputation techniques we have handled non-responding in various ways before analysis. One option was to only include a participant if a certain percentage, e.g. 80%, of the entire number of replies during the follow-up period was answered. Another alternative was to define a certain period of particular clinical importance where replies must be given in order to avoid exclusion. In this commentary, the results from the following alternatives are reported; a) the full data set, b) respondents with a minimum of 80% response rate and c) those answering all the first eight weeks, respectively. The latter was chosen as these patients were included in the studies while experiencing an episode of LBP, and thus the eight first weeks of the trial were chosen as the period of the most interesting development.

### Research questions with appropriate analytical methods, results and discussions

We have raised some research questions that are relevant to researchers in the area of musculoskeletal pain and illustrated appropriate analytical methods with which to answer them. We have started with the very basic, descriptive analyses. Then, specific time points were selected for analyses. As a third step, a specific event was selected but time was allowed to vary, and in the fourth approach, both time and event may vary. Finally, the existence of subgroups was the basis for the last set of analyses.

#### How many days with pain do patients experience?

##### *A: What is the total number of pain days?Table* 1

**Table 1:** DESCRIPTIVE measures, variable- oriented hypotheses

**Research question**	**Outcome**	**Method of analysis**	**Results from the model data set**	
			**All respondents, n = 244**	**Highly compliant respondents, answering 80% (≥15/18 weeks), n = 161**
1A: Crude outcome	Total number of 1: days with pain from 18 weekly measures, 2: weeks reported	Summaries.	1: Mean 33.0, Range 0 – 126 Short duration: Mean 24.5, Range 0-124 Long duration: 41.1, Range 0-126 2: Mean 15.2. Range 2-18	1: Mean 36.4. Range 0-126 Short duration: Mean 27.4, Range 0-124 Long duration: mean 45.4, Range 4-126 2: Mean 17.3, Range 15-18
1B: Difference in weekly outcome between groups	Average number of pain days per week	Student’s *t*-test	Short duration: 1.6 Long duration: 2.8 p < 0.001	Short duration: 1.6 Long duration: 2.6 p < 0.001
2A: Proportion with different levels of the condition	Incidence of recovered = reporting 0 pain days week by week	Proportion, i.e. percentage of subjects who are recovered compared to those who are not	Illustrated in Figure 1a Proportions recovered week 8: Short duration: 58.6% Long duration: 30.7% OR = 3.19 (1.88 – 5.72) RR = 1.8 (1.62 – 1.93) Long duration reference category	Illustrated in Figure 1b Proportions recovered week 8: Short duration: 58.3% Long duration: 32.5% OR = 2.91 (1.50 – 5.65) RR = 1.66 (1.51 – 1.83) Long duration reference category
2B: Incidence at a prespecified time point	Proportion of patients recovered = reporting 0 or 1 pain days at the chosen time, e.g. the 5th week	Logistic regression (or other generalised linear regression models)	Short duration: 58.7% Long duration: 27.7% OR = 3.71 (2.1-6.6) RR = 1.75 (1.4 – 2.3) Long duration reference category	Short duration: 58.2% Long duration: 28.0% OR = 3.58 (1.8 - 7.0) RR = 1.72 (1.34 – 2.3) Long duration reference category

It has been suggested that the total number of days with back pain over a period would be a good way of assessing chronicity [13]. With weekly data collected prospectively, accurate data summaries are now feasible**.** In the model sample, the total number of days with LBP ranged from zero to 126, with a mean of 33.0 days. When analyzing the high compliers only, the mean was slightly higher.

##### *B: What is the average number of pain days per week? Table* 1

The individual means were calculated as sum of reported pain days divided by the number of weeks the individual actually reported data. When analyzing the subset of high compliers, the estimate of the group with long duration changed marginally.

##### *Comments*

Generally, summary outcomes are easy to interpret and give clinically meaningful estimates, and summarizing weekly data may indeed distinguish patients with a more persistent course. Summary scores could also be used as an outcome in multivariate models. On the other hand, the simplification of data that is the core feature of the summary statistics ignores course patterns and time to improvement. Potentially important differences could be missed e.g. would the sum score during ten weeks be equal for a patient having pain level 3 during all weeks and a patient with five weeks of “pain 6” followed by five weeks of “pain 0”.

#### *What is the proportion of participants “recovered” at a specific time point?*

##### *A: Incidence of recovery week by week. Table* 1*& Figure* 1

**Figure 1:**
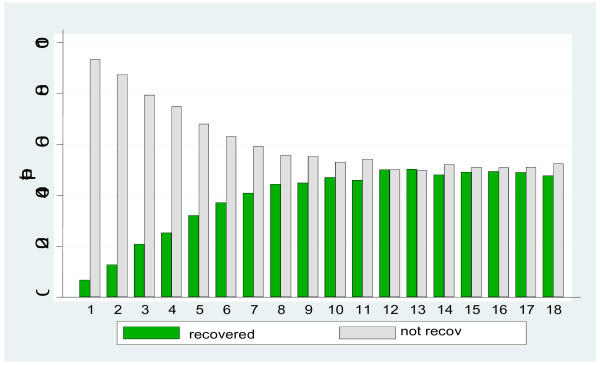
Percentage of patients recovered (LBP days = 0) and unrecovered in each of 18 weeks following a first visit to a chiropractor (n = 212 week one; n = 186 week 18).

To describe the between-individual variance of the population, one possibility is to present the proportion that meet a criterion of interest at different time points. In our studies, recovery at different time points was studied. Thus, a dichotomised outcome “recovered “(defined as reporting zero days with LBP [14]) and “not recovered” was used for each week throughout the study. Comparisons of the proportion of recovered between groups can be done as in section B below, but this analysis should be applied only to a pre-specified time point of interest to avoid the problem of mass-significance due to multiple testing [15]. As can be seen in Figure 1, the proportion of respondents who recover increased up until week 8 to about 50% of the population, and remained more or less stable thereafter. For patients with < 30 and > 30 days LBP last year, 58% and 31% of the populations respectively had recovered after eight weeks (results not shown). The high compliers had a recovery pattern very similar to that of the entire population (results not shown).

##### *B: Incidence of recovery at a prespecified time point, Table* 1

Repeatedly collected data can also be used in ordinary logistic regressions choosing a specific time point as the time of interest (to patients, clinicians or to third party payers), i.e. not selecting time points on the basis of Figure 1 above. According to previous studies of patients with LBP in primary care, many patients report improvement by the fourth to fifth week after consultation [16]. Recovery (this time reporting zero or one day with pain [17]) was the event and five weeks was chosen as our time point of interest. The proportion of patients recovered at this point in time was compared for those with long and short previous duration. Note that this approach could easily accommodate more predictive variables. The result from the logistic regression analysis showed that the odds ratio for recovery at week 5 was significantly higher for patients with a short previous duration compared to those with a long previous duration (OR = 3.71, 95% CI: 2.1-6.6). Another outcome parameter is the risk ratio, RR, (using a logarithmic link function instead of the logit function in logistic regression) of 1.75(CI: 1.4-2.3). When analyzing the high compliers only, the estimates changed marginally.

##### *Comments*

Defining recovery as the event of interest, maybe “relief rates” would be a better term than hazard rates, as the outcome parameter. Using this outcome, the numbers and proportions of patients with a successful course can be estimated, and also the numbers needed to treat can be calculated. However, individual trajectories are ignored at the expense of population overview.

#### *What is the time to recovery?*

##### *A: Incidence of “recovery” throughout the study period, Table* 2*& Figure* 2

**Table 2:** INCIDENCE measures, variable- oriented hypotheses

**Research question**	**Outcome**	**Method of analysis**	**Results from the model data set**	
			**All respondents, n = 244**	**Highly compliant respondents, answering 80% (≥15/18 weeks), n = 161**
3A: Incidence during the full study period for the whole sample and for subgroups	Recovery, i.e. reporting 0 or 1 pain days in 2 consecutive weeks = Event	Time to event analysis, with Kaplan Meier curves. Log rank test for differences between groups	Illustrated in Figure 2. Logrank test for effect of previous duration: p < 0.001	Logrank testfor effect of previous duration: p = 0.002
3B: Incidence for the full study period in relation to the selected predictive variables	Recovery, i.e. reporting 0 or 1 pain days in 2 consecutive weeks = Event	Time to event analysis with a) Cox proportional hazard regression or b) Discrete hazard regression	Hazard ratio (HR) showing recovery, long duration reference, estimate and 95% CI: a) 1.95 (95% CI: 1.4-2.6), b) 2.03 (95% CI: 1.5-2.7).	Hazard ratio (HR) showing recovery, long duration reference, estimate and 95% CI: a) 1.95 (95% CI: 1.4-2.6), b) 2.03 (95% CI: 1.5-2.7).
3 C: Time point for an event during the pain course	The time point of change in the course of pain = Event	Spline regressions, the event defined as the intersection of linear regression lines (the knot).	Short duration: knot at 4.5 weeks Long duration: knot at 5.9 weeks	Short duration: knot at 4.4 weeks Long duration: knot at 5.8 weeks

**Figure 2:**
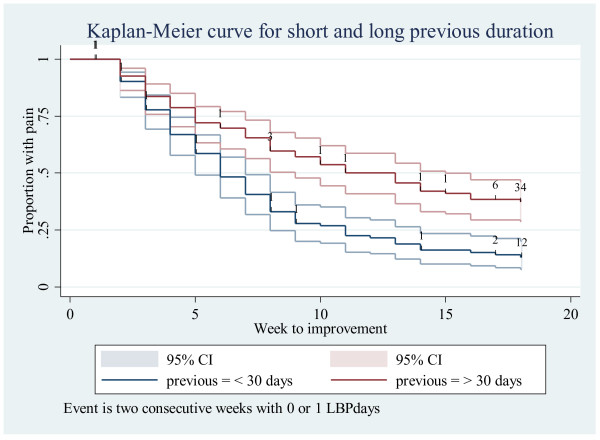
Incidence of “recovery” (reporting zero or one pain days in two consecutive weeks), stratified by previous duration (> 30 days in pink, < 30 days in blue) for the full data set.

Using repeated measures, it is possible to monitor a population at risk to study the incidence of an event over time, that is, an extension of the approach in section 2 above. To our knowledge, this type of data has thus far been unobtainable and provides a “true” incidence of the event studied. When exploring different conditions/variables, the event will be defined according to clinical/medical or other parameters.

In survival analysis, one of the main application areas for this kind of analysis, mortality was the event under study. As we would expect the patients to get better, we studied the positive event “recovery”, for this example data set defined as zero or one pain day reported in two consecutive weeks [18]. Our illustrated analysis can be described in general terms as a time-to-event analysis, using techniques and notions that emanate from survival analysis [19]. Kaplan-Meier curves are one of the basic descriptive tools to summarize outcome over time. Figure 2 displays the number of reported events in a Kaplan Meier curve, i.e. the incidence of patients recovering. The logrank test for differences in the rate of recovery between the long and short duration groups was significant (p < 0.001). The same analysis was performed for the high compliers (curve not shown). Again, the logrank test showed significant differences in the rate of recovery between the two groups (p = 0.002).

##### *B: Incidence of “recovery” in relation to predictive variables, Table* 2

Kaplan-Meier curves stratified by a predictor variable and a test for difference between categories of the predictor were the starting point for this analysis. More elaborate analysis allowing for several predictor variables can be done with Cox Proportional Hazard regression [19] with time (week)treated as a continuous variable. In our data set, it could be hypothesized that the risk of future LBP would be predicted by past LBP. If the risk of the condition studied changes between groups of subjects over time, the assumption of proportional hazards might be violated and should be adjusted for. When the outcome is measured at discrete time points (e.g. only a few weeks observed), a discrete hazard regression analysis [19] could be more appropriate than the Cox model. In our model data, a test of the proportional hazard assumption showed that the assumption was not rejected. The hazard ratio (HR = 1.95) showed significant differences (95% CI: 1.4- 2.6) between the two groups, meaning that the group with a short previous duration had nearly double the “risk” of the studied event recovery. Performing the analysis for the high compliers only did not change the estimate.

When the outcome event was considered to be measured at discrete time points and no assumptions were made of proportional hazards, a discrete hazard regression analysis showed a statistically significant hazard ratio of 2.03 (95% CI: 1.5-2.7) between the groups, again pointing towards the patients with a shorter previous duration of LBP having the best chance of recovery during the study period. Performing the analysis with the high compliers only did not change the estimate.

##### *C: When does a particular event occur at an individual level? Table* 2

Having access to repeatedly observed data, detailed changes in the course of the condition can be studied. Concerning LBP, it is often observed that the patient’s course is different in a first phase up to approximately the fourth or fifth week with a rapid recovery [7,16], and then a much slower improvement is observed [20]. The shift in the course may be estimated more precisely by applying a spline regression technique where two regression lines are fitted to describe the two sections of the course and the intersection (“knot”) between these regression lines estimates the shift in the course. This application of the spline regression technique is also known as a piecewise linear regression. The spline regression can be applied for the whole group, for pre-defined subgroups of interest such as those with long or short previous durations, and also for each individual separately provided that the number of weekly observations is sufficient (as described in section 5B below). In the model data set, the regression lines for short and for long previous duration were clearly separated, with statistically significant differences in three of the four parameters that define the spline regression lines. The patients with a short previous duration had a course change at 4.5 weeks, compared to the patients with a long previous duration, who had a later course change at 5.9 weeks. Analyzing the high compliers separately only marginally changed the estimates.

##### *Comments*

Looking at incidences by means of hazard ratios yields interpretable results in terms of proportions recovered and holds the possibilities of the analytic methods used for survival analyses [19]. Recovery from LBP may be defined more or less stringently concerning pain days and period. In a condition like LBP it is, however, problematic to define recovery as the “event” since patients often experience fluctuations in the condition or recurrences following a pain free interval [21]. The possibility of repeated or recurrent events is not dealt with in the standard implementation of survival analyses.

Further, the definition of event will influence the Kaplan-Meier curve. Had we defined recovery as four consecutive weeks with little or no pain (instead of two weeks), a smaller number of patients would have accomplished this and thus the curves would have been more horizontally oriented.

Throughout, regardless of the outcome parameters, the type of data (continuous or count) and the level of compliance, the group with a short previous duration of LBP had significantly higher “risks” (chance) of recovery. This suggests that for our model data set, the methods were robust as the conclusions pointed in the same direction regardless of method. Further, incidences over the full study period as well as at a specific point in time can be calculated from the full data set, as including poor compliers only marginally changed the estimates. In other data sets, this may, however, not be the case. In the mentioned Swedish study [4], poor compliers were found to have a less positive development of their LBP over time, which is why we suggested that the effect of high compliance should be evaluated.

Finally, we also illuminated a method to estimate the point of change in a course of pain using spline regression analysis. Again, a difference between the two groups was noted, and the use of only high compliers did not change the estimates to any large degree. Spline regression is, of course, an approximation of the true, rather fluctuating, course of pain during the 18 weeks. The specification of the spline regression has, however, been done with those few parameters that were of vital clinical interest. To capture all the features of the course of pain would surely need several more parameters and such an approach would probably lose clinical interpretability.

#### *How is the repeatedly measured data associated with baseline (predictor) variables?*

##### *Variation in events over the whole time period. Table* 3

**Table 3:** LINEAR MODELS, variable- oriented hypotheses

**Research question**	**Outcome**	**Method of analysis**	**Results from the model data set**	
			**All respondents, n = 244**	**Highly compliant respondents, answering 80% (≥15/18 weeks), n = 161**
4A: Association of baseline variables with outcome	Weekly recorded pain days, count variable, assuming a binominal distribution	Multilevel mixed-effects logistic regression or generalized estimating equation assuming a logit link function (Long previous duration reference category)	Subject specific OR = 3.31 (95% CI: 2.1-5.1) Population average OR = 1.96 (95% CI 1.4-2.6) (Note: Interaction duration*week significant)	Subject specific OR = 2.67 (95% CI: 1.6-4.5) Population average OR =1.52 (95% CI 1.1- 2.2) (Note: Interaction duration*week significant)
4B: Association of baseline variables with outcome	Weekly recorded pain days, count variable, assuming a Poisson distribution	Multilevel mixed-effects Poisson regression assuming a log link function (Long previous duration reference category)	Subject specific IRR = 1.92 (95% CI: 1.5 – 2.4) (Note: Interaction duration*week significant)	Subject specific IRR = 1.82 (95% CI: 1.4 – 2.4) (Note: Interaction duration*week significant)
4 C: Association of baseline variables with outcome	Weekly recorded pain days, considered a count variable and assuming a normal distribution	Generalized linear regression or mixed linear model assuming an identity link function	Average difference in pain days for Long duration – Short duration 1.20 (95% CI: 0.8 – 1.5) (Note: Interaction duration*week significant)	Average difference in pain days for Long duration –Short duration 0.95 (95% CI: 0.6-1.4) Note: Interaction duration*week significant)

In these examples, we examined the association of the baseline variable “previous duration” with the outcome “number of pain days”. Throughout, the effect of time is considered a fixed effect (to account for systematic differences between weeks and to obtain estimates for each separate week). All models in this section are statistically and computationally more advanced than those in the previous sections. This whole area of statistical models has expanded very much during the last 10–15 years thanks to theoretical advancements as well as the development of suitable software. It is outside the scope of this article to give details here, we recommend texts such as those by Twisk [22] and Rabe-Hesketh [23].

A: This approach used either a multilevel mixed-effects logistic regression or a Generalized Estimating Equation (GEE)[24] to obtain an effect parameter as a subject-specific Odds Ratio (OR), or a population average OR, the former from the multilevel model, the latter from the GEE analysis. Both models are extensions of linear models to accommodate repeated data. The distribution of the outcome was assumed to follow a binomial distribution and a logit link function described the relation between the outcome and the predictor or baseline variables. The argument that the number of days will follow a binomial distribution was that the respondents evaluated a yes/no reply for each day when answering the weekly measure, really asking themselves “Did I have pain on Monday? On Tuesday?” etc. before summing up. Modeling the covariance structure of the correlated repeated measurements, the associations of the baseline variables as well as possible interactions of such variables with the outcome can be studied in several different ways. The outcome, number of pain days, was considered a count variable. The patients reporting short duration pain the previous year had significantly less odds of reporting pain during the study period. Calculating the subject specific odds ratio (OR = 3.31, 95% CI: 2.1-5.1) indicated the odds for a single subject, whereas the population average (OR = 1.95, 95% CI: 1.4-2.6) gave an estimate for the “averaged” subject closer to unity than the subject specific odds ratio as expected when the two approaches of estimation were considered. Performing the analysis for the high compliers only lowered the estimates somewhat for both subjects and population.

B: As above, the outcome was considered a count variable, but a Poisson distribution was assumed instead of a binomial distribution. The multilevel analysis showed a significant difference (Incidence Rate Ratio, IRR = 1.92, 95% CI 1.5-2.4) between the patients with a short previous duration of pain compared to those with a long previous duration, indicating that the former had lower odds of reporting many pain days. The estimate was lowered somewhat when analyzing the high compliers only. In this case, a multilevel Poisson regression [18] was appropriate. Our outcome “number of pain days” may not seem to be an obvious candidate for a Poisson distribution, because of the upper limit of seven days each week since a restriction of this kind is not appropriate for a Poisson distribution. We have added the Poisson analysis just to show the appropriate method for another type of outcome such as “number of times of taking pain medication” during the week, which in theory may have no or at least a very high upper limit.

The outcome parameter is an Incidence Rate Ratio (IRR). A property of the Poisson distribution is that the mean and the variance are equal. In some applications this may not be the case, and in particular the variance can be greater than the mean, which is referred to as a case of over dispersion. Then an analysis using a negative binomial distribution may be appropriate (not described here, see [24]).

C: Considering “days with pain,” a continuous outcome may not be the obvious choice either for our model data set, as the outcome variable “number of pain days” was discrete and had an upper limit of 7. In the investigation of other conditions, the outcome could be continuous. If so, the association with baseline variables can be studied using mixed linear models. In our example, an autoregressive covariance model was chosen assuming decreasing correlation with increasing time and confirmed with Akaike’s Information Criterion [25], and the associations of baseline variables as well as the interactions of these variables with the outcome were studied. It should be noted that the other available baseline variables besides previous duration were included in the model as well (not presented here). In mixed linear models, with time as the explanatory factor, the association of previous duration and weekly pain days showed a significant difference for patients reporting long and short previous durations (average difference in pain days 1.20 (95% CI: 0.8 – 1.5)).

Performing the analysis for the high compliers lowered the estimate, but did not change the significance. Including more baseline variables in the model did not affect the parameter estimates noticeably (analysis not shown).

##### *Comments*

These methods are designed for repeated measures and take correlation between outcomes measures and different time points into account. In this way the richness of the data is maintained and trustworthy significance levels are achieved. However, the results may sometimes be more difficult to interpret in a clinically meaningful way. The models are statistically more sophisticated, require more work for the specification of the analyses, but they are implemented in standard statistical software (such as SPSS, STATA, SAS) and our analyses and results are based on these softwares.

With GLM it is possible, as demonstrated, to use both count and continuous variables, and the outcome could be binomially, Poisson, or normally distributed. In our model data, regardless of model, the results all pointed in the same direction. We conclude that for our chosen variable and outcome, the choice of method may not have been utterly important. However, because our repeated outcome was most accurately classified as a count variable following a binomial distribution, we trusted the estimates from the generalized estimating equation model and the corresponding multi-level model to be the most valid estimates.

A final note has to be added to the results of this section about the significant interaction found between previous duration and week, the latter variable representing time under observation.

This implied that a second step in the analysis was necessary to fully understand how the pain course developed over time for the two duration groups. In the present context with examples and suggestions for analysis this step is not further elaborated.

#### Are there subgroups of patients with similar courses of pain within the studied population?

There are several methods that are useful when looking for patterns within repeated data using person-oriented approaches. The examples below range from purely descriptive (A) which rely on a clinical impression, through hierarchical methods (B) which are mathematical in origin but requires a supplementary clinical judgment, to the very mathematical methods (C) which rely on the acceptance of a pre-specified statistical model.

##### *A: Visual description, Tables* 4 & 5

**Table 4:** SUBGROUPS, person-based hypotheses

**Research question**	**Outcome**	**Method of analysis**	**Results from the model data set**		
			**All respondents, n = 244**	**Respondents answering ≥ 80%, n = 161**	**Respondents answering all first 8 weeks, n = 133**
5: Are there subgroups of patients?	Subgroups as clusters with low within-cluster variation and high between-cluster variation	A. Visual inspection based on plots of the course of pain in a graphical presentation where predefined criteria of directions in early and late phases, a qualitative approach	A: Illustrated in Table 5	Not applied	Not applied
		B: Regression coefficients from spline regression (1 knot) derived from each subject were used in Wards’ hierarchical cluster analysis. Optionally this analysis was followed by K-means cluster analysis. Inspection of number of clusters based on the Calinski-Harabasz criterion and the criteria by Duda & Hart	B: Not done due to lack of degrees of freedom in spline regression of some individual subjects	B: 4 clusters suggested.	Not applied
		C: Wards’ hierarchical cluster analysis, optionally followed by K-means cluster analysis, applied directly on the weekly number of pain days for the first 8 weeks. Cluster criteria as in B.	Cluster 2: 79	Percentage with short duration in these clusters:	C: 6 clusters suggested. Percentage with short duration the previous year in these clusters:
			C: Not applied	Cluster 1: 39	Cluster 1: 58
				Cluster 2: 49	Cluster 3: 20
				Cluster 3: 85	Cluster 4: 33
				Cluster 4: 37	Cluster 5: 52
				C: Not applied	Cluster 6: 50

**Table 5:** Categories used to classify individual pain patterns by visual analysis

**Possible categories describing the entire course by visual analysis**	**Individuals in each subgroup, n, (%)**	**Total number of pain days in each subgroup, mean (sd)**	**Percentage with short duration the previous year**
Improved-Mainly recovered	25 (12)	8.92 (6.01)	76
Improved-Stays in the category	64 (30)	26.83 (14.65)	33
Improved-Fluctuating	23 (11)	37.43 (21.17)	30
Improved-Moves towards mainly worsened	2 (1)	95.00 (11.31)	0
Unchanged-Mainly recovered	13 (6)	5.54 (5.68)	54
Unchanged-Moves towards mainly improved	18 (8)	38.11 (21.81)	50
Unchanged-Stays in the category	10 (5)	45.60 (51.21)	33
Unchanged-Fluctuating	40 (19)	51.28 (30.95)	38
Unchanged-Moves towards mainly worsened	2 (1)	84.00 (29.70)	0
Worsened-Mainly recovered	1 (0.5)	12	0
Worsened-Moves towards mainly improved	2 (1)	37.5 (17.68)	50
Worsened-Fluctuating	13 (6)	55.00 (20.29)	0
Worsened-Stays in the category	2 (1)	111.5 (17.68)	50

The first step is to categorize the pattern in relation to the early course (improved, unchanged or worsened). Afterwards that category is combined with the relevant late course.

The course of a disease (LBP in our model sample) can be described by a graphical representation of each participant’s development over time, in this case the number of pain days by weeks. In studies with relatively few individuals (up to about 200 participants) it is possible to analyze such courses by hand by describing the characteristics of the pain courses including when the changes take place; thus attempting to identify groups of patients with similar pain patterns. The inter-observer reliability of such a visual evaluation was found to be substantial (kappa = 0.7) between two observers analyzing 78 courses [7]. Depending on the level of detail in the visual description this may result in very few subgroups or in as many subgroups as the number of individuals studied. Therefore, we suggest making clinically relevant a-priori definitions of the subgroups to be looked for in order to improve the objectivity and reliability of the visual descriptions. The method relevant for the categorization of course patterns will depend on the known clinical characteristics of the investigated disease and the type of population. Our approach to visually describing the pain patterns was performed in two stages as described earlier [7]. In short: The development of an early course (weeks 1 – 4) and a later course (week 5 and later) was defined pre-hoc. It may be relevant to split other data sets differently depending on the condition studied and the length of the follow-up. Visually described patterns could be derived from the text message responses of 215 patients. The analyses was not performed if data are missing for more than two weeks in a row, in which case the subject had to be excluded.

The combination of the categories for the early and the late course resulted in 13 possible categories. All the 13 categories were represented in the model data set, consisting of from one (0.5%) to 64 (30%) patients (Table 5). Patients described as recovered had the fewest LBP days and patients classified as “worse” in the late course had the most LBP days. Further, the patterns with recovery or lasting improvement had a larger proportion of patients who had < 30 LBP days the previous year than patterns of fluctuation or worsening (Table 5).

##### *B: Cluster analysis using an hierarchical method*

**Figure 3:**
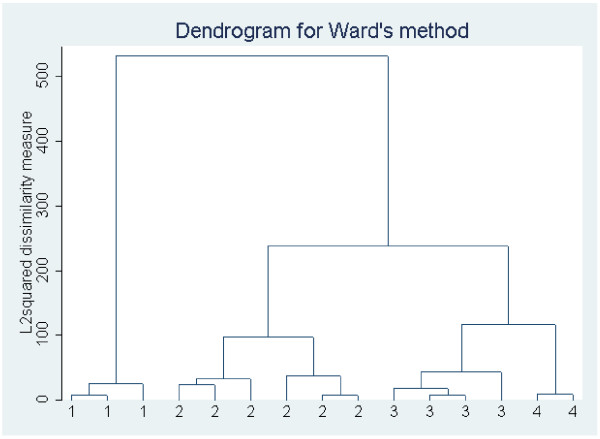
A dendrogram obtained with Ward’s method, describing the formation of clusters.

The trend course of the outcome variable over the study period can be explored with cluster analysis to search for subgroups. The simplest alternative would be to use all individual 18 weekly data points as cluster parameters, but this is practically difficult both with respect to the burden of heavy computations and of missing data. Therefore, the individual courses in our model sample were summarized with different mathematical approaches, limiting the number of cluster parameters. The parameters were then used in a hierarchical cluster analysis, Ward’s method [26], to reveal subgroups. Ward’s method resulted in a dendrogram (Figure 3), a graphical representation of the cluster building process, which was then scrutinized visually to find the optimum number of clusters, together with a mathematical criteria such as Caliński -Harabasz [27] or Duda Hart [28]. Then, the two best solutions suggested by the Caliński -Harabasz criterion were explored in terms of detail vs. overview to find the optimal final solution. Thus, in approach a), the Caliński -Harabasz criterion suggested that the four-cluster solution was optimal. For the method applied in b), the Caliński -Harabasz criterion suggested a six-cluster solution.

a) Each course was described by two regression lines describing the early trend and the later trend, respectively. Using a spline (nonlinear regression) technique, the intersection between the two was then calculated. From these analyses, four parameters described each profile: the intercept and slope of the early trend, the difference in slope between the early and late trend and the intersection between the two regression lines. These four parameters were then used as cluster parameters. Note that the regression parameters serve to approximate the weekly variation in the data, and they must be evaluated with respect to the degree of fit for each patient.

Cluster 1 (28 individuals): This subgroup contained the oldest individuals (mean age 47 years), who reported the highest number of total pain days (50 days).

Cluster 2 (68 individuals): This subgroup contained the youngest individuals (mean age 42 years), who reported 33 pain days throughout.

Cluster 3 (20 individuals): The patients here were mainly male (70%), reported the most leg pain (40%), reported least pain days (20 days) and most (85%) had had short previous duration of LBP.

Cluster 4 (45 individuals): This subgroup contained the largest proportion of patients (63%) that had had long previous duration of LBP and reported 36 days of pain throughout.

b) Each course was described by the eight first weekly measurements only. As mentioned earlier, this was considered the period where the most change in pain days is likely to be noticed in our population. Thus, in this cluster analysis, eight cluster parameters were used.

Cluster 1 (12 individuals): This subgroup had many women (67%) and reported 37 pain days throughout.

Cluster 2 (24 individuals): This subgroup had the youngest patients (mean age 40 years), mainly women (67%) and reported 35 pain days throughout.

Cluster 3 (15 individuals): This group had mainly patients with long previous duration of pain (80%), and reported 58 days of pain throughout.

Cluster 4 (28 individuals): These patients were mainly male (64%), and most (79%) reported short duration of previous pain. They had 21 pain days throughout.

Cluster 5 (46 individuals): This group reported fewest days of pain, only 13.

Cluster 6 (8 individuals): This was the subgroup with the oldest individuals (mean age 49 years), they reported most leg pain (50%)and had the highest number of pain days, 101 in total.

##### C: *Other exploratory approaches*

The cluster analysis shown here is just one of several different alternatives that can be used for this and similar data sets. An excellent overview of cluster methods in theory and in applied research is found in Everitt BS, “Cluster Analysis”[26]. Among these methods are those using finite mixture densities with a range of approaches including mixtures for multivariate normal distributions as well as mixtures for categorical data (latent class analysis) and Bayesian analysis of mixtures.

##### *Comments*

Several other possibilities also exist for aggregating data points into useful descriptions of the course suitable for cluster analysis. Adding to the linear regression, a second or third degree regression can be used to also approximate the course. Similarly, the spline function can be extended to contain two or more knots. Clinical judgement should be used to evaluate what is relevant for any particular condition. We have previously argued that to secure solid course estimates, only highly compliant responders (arbitrarily defined as those answering more than 80% of the time) should be used in these kinds of analyses (in a))[20] and extending the argument, only those with a full response when using the crude data as parameters (b).

Further, in both A and B, the subgroups/clusters formed in this mathematical way should be examined for clinical meaningfulness. For instance, the available clinical baseline variables associated with each cluster can be tested for difference between clusters.

We have illustrated possible ways of exploring the model data set for subgroups based on the repeated data. Concerning non-specific LBP, our approach was based on the hypothesis that patients with the same “category” of LBP might exhibit a similar clinical course. The visual description of individual’s pain patterns was a pragmatic and clinically meaningful way to distinguish between obviously different patient profiles, but the method is time consuming and only doable in small samples. However, the choice of subgrouping methods is, as for all analyses, dependent on which assumptions of the data and their distributions the researcher is willing to accept.

A number of data mining approaches can be used for pattern recognition instead, e.g. cluster analysis, latent class analysis, artificial neural networks, and probabilistic data mining. It is beyond the intents of this commentary to test, describe and compare these.

## Conclusions

When new methods of data collection are introduced, it is always pertinent to consider the possibilities, advantages, implications and challenges this entails. Making use of a technology available to a majority of people in the modern world, mobile phones, doors are opened to repeated measurements from large populations.

Having access to repeated data, it is not self-evident what methods of analysis to use. We have intended to give an overview of some approaches to analyses considered by our group of researchers, but other relevant methods exist and, possibly, different data from other research areas may require yet different methods. In this commentary, the methods are presented very briefly, and we encourage readers to use the references included for a deeper understanding.

Ultimately, the choice of analytic approach will depend on the following questions: what is the research question to be answered, what kind of data is the outcome variable, what distribution does it have and what is the within-subject correlation? The answers will determine the most appropriate method of analysis.

In analyzing repeated data, the issue of within-subject correlation can be avoided by aggregating the individual measures into a summary measure prior to analysis. However, information on individual variation is then lost, resulting in over-simplification. Thus, methods of analysis that account for this covariance may be the most appropriate.

In our model data, patients with > 30 days LBP the preceding year consistently demonstrated an increased risk of a “poor prognosis” compared to those with < 30 pain days in all the variable oriented analyses. Further, different patient profiles could be identified based on the pain trajectories emerging from cluster analyses of the frequently repeated outcome measure. Thus, it seems that repeated measures can be analysed in several meaningful ways with both traditional analytic approaches using standard statistical packages, as well as recently developed statistical methods that will utilize all the vital features inherent in the data.

## Competing interests

The authors declare that they have no competing interests.

## Authors’ contributions

IA was involved in the design of this commentary, participated in the analyses and wrote the first manuscript draft. LB was responsible for the analyses. AK and NW were involved in the design and analyses of the data. IJ and GB were supervising the study process. All authors were involved in finalizing the manuscript.

## Pre-publication history

The pre-publication history for this paper can be accessed here:

http://www.biomedcentral.com/1471-2288/12/105/prepub
